# Unveiling and verification of mitochondria-related genes as potential diagnostic biomarkers in ulcerative colitis based on bioinformatics analysis and experimental validation

**DOI:** 10.1371/journal.pone.0336224

**Published:** 2025-11-04

**Authors:** Hongliang Chen, Ning Li, Xuerong Liu, Mingyu Ran, Xinyu Geng, Jihan Qi, Jiawei Qiu, Xueyu Cang, Shiling Huang, Yingying Tian, Ram Prasad Chaulagain, Shizhu Jin

**Affiliations:** 1 Department of Gastroenterology and Hepatology, Second Affiliated Hospital of Harbin Medical University, Harbin, China; 2 Department of Neurology, Second Affiliated Hospital of Harbin Medical University, Harbin, China; 3 Department of College of Bioinformatics Science and Technology, Harbin Medical University, Harbin, China; OMICS, PERU

## Abstract

Immune dysregulation is a pathogenic factor in ulcerative colitis (UC), in which mitochondrial involvement is increasingly recognized. This study aimed to construct a diagnostic model using mitochondria-related genes, identify new target genes, and illuminate the roles of mitochondria-related genes in energy metabolism, immune infiltration and the pathogenesis of UC. RNA expression data from 465 patients with UC and 154 healthy controls (HCs) were obtained from the GEO database. A total of 128 mitochondria-related differentially expressed genes (Mito-DEGs) were identified between patients with UC and HCs. A diagnostic model constructed from 20 genes showed satisfactory discrimination, calibration functions, clinical benefits and clinical impacts. Enrichment and immune infiltration analyses revealed significant differences in mitochondrial structure and function, immune cell disorders, and signaling pathway activation between the high- and low-mitochondrial gene expression UC groups. Correlations between mitochondrial structure and function and immune cells were evaluated. Single-cell RNA sequencing data were used to analyze the hub gene distribution, cell‒cell communication, and enrichment. Cell‒cell communication analysis revealed that immune response and pathogenesis pathways are activated in UC. Experiments revealed that the expression of the ACADM and ACADSB genes was decreased in UC patients. Mitochondrial dysfunction contributes to the pathogenesis of UC by altering energy metabolism, promoting immune disorders and activating pathogenic signaling pathways. The mitochondria-related genes are valuable for the diagnosis of UC. ACADM and ACADSB may play important roles in UC pathogenesis.

## Introduction

The incidence and the prevalence of ulcerative colitis (UC), an inflammatory bowel disease with a relapsing and remitting course, have increased rapidly worldwide [1]. Currently, the mutual effects of genetic factors, dysregulated immune responses, the environment and disturbances in the intestinal flora are all considered pathogenic factors of UC [2]. However, the underlying mechanisms of UC remain largely unknown.

UC progression involves chronic inflammation driven by immune dysfunction [3]. Studies have shown that UC is characterized increases in the levels of innate immune cells, including neutrophils, macrophages, dendritic cells and monocytes [4,5]; a decrease in the diversity and maturation of B cells [6]; and the dynamic remodeling of colonic CD8^+^ T cells [7]. Although the immunopathogenesis of UC involves complex signaling pathways [8], the regulatory factors involved are still not fully understood.

Mitochondria are dynamic organelles that regulate critical cellular processes including ATP production, metabolite synthesis, and calcium homeostasis [9]. Our previous study revealed that mitochondrial dysfunction is a key factor with inherent susceptibility in the development of UC [10], that may drive pathogenesis by causing metabolic defects in the epithelial cells of the colon, altering cell phenotypes, and promoting the release of mitochondrial damage-associated molecular patterns [2]. As complex organelles, mitochondria are involved in numerous signaling pathways [11], but whether they promote the development of UC through immune dysfunction is unclear.

**Fig 1:**
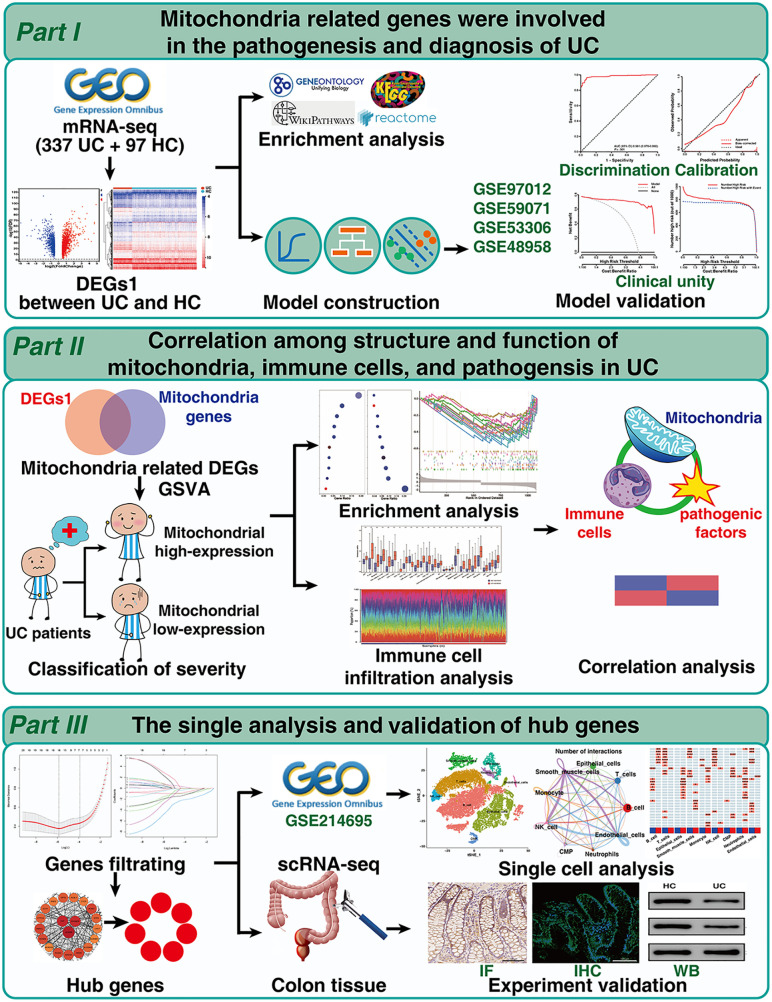
Workflow of the study.

In this study, mitochondria-related differentially expressed genes (Mito-DEGs) were identified by comparing samples from UC patients and healthy controls (HCs) and a satisfactory diagnostic model was constructed and validated. To explore how mitochondria are involved in UC pathogenesis, we divided UC samples into high- and low-mitochondrial gene expression groups, and performed immune cell infiltration, enrichment, and correlation analyses. Hub mitochondria-related genes involved in fatty acid metabolism were subsequently identified via single-cell analysis, and the downregulation of acyl-CoA dehydrogenase medium chain (ACADM) and short and branched chain-specific acyl-CoA dehydrogenase (ACADSB) in UC patients was experimentally validated. A flowchart of the study is presented in Fig 1.

## Materials and methods

### Acquisition and preprocessing of datasets

RNA sequencing data were obtained from the Gene Expression Omnibus (GEO). A total of 11 datasets (465 patients with UCs and 154 HCs) were used in this study and were grouped into a training set and 4 validation sets (S1 Table in S1 File). The “ComBat” algorithm was used to correct for batch effects in the GEO datasets. Robust multichip averaging analysis was performed on the microarray data, and log2-transformation and normalization were performed to make the gene-expression profiles obtained by different platforms comparable. A total of 1136 mitochondria-related genes were identified from MitoCarta3.0 [11].

### Identification of differentially expressed genes (DEGs)

The “limma” R package was used to identify DEGs between patients with UCs and HCs and DEGs between UC patients with high and low expression of mitochondria-related genes with a threshold of |log_2_ (fold change) | ≥ 2 and false discovery rate (FDR) < 0.05. The “VennDiagram” R package was used to identify the Mito-DEGs.

### Functional enrichment analysis

The “GSVA” R package and the identified Mito-DEGs were subsequently used to calculate risk scores, and UC patients were subsequently divided into high and low mitochondrial gene expression groups on the basis of their median risk scores. To explore the significantly enriched molecular pathways and biological processes based on the transcriptomic data, gene set enrichment analysis (GSEA) was performed using the gene sets “c5.go.v2023.1.Hs.symbols”, “c2.cp.kegg.v2023.1.Hs.symbols”, “c2.cp.reactome.v2023.1.Hs.symbols”, and “c2.cp.wikipathways.v2023.1.Hs.symbols” from the Molecular Signatures Database [12]. Gene Ontology (GO), Kyoto Encyclopedia of Genes and Genomes (KEGG), WikiPathways (WP) and Reactome (REAC) analyses were performed on the DEGs via the R package “clusterProfiler”, with an FDR < 0.05 considered significant.

### Immune cell infiltration analysis

Single-sample gene set enrichment analysis (ssGSEA) [13] and xCell [14] were used to convert individual sample gene expression data into matrices of immune cell gene sets. 28 immune cells were included in the ssGSEA, and 64 immune cells were included in the xCell. These two methods were employed to evaluate immune cell infiltration status in UC patients with both high and low mitochondrial gene expression, and the results were visualized with the “ggstatsplot” and “cowplot” R packages.

### Correlation analysis

Pearson’s correlation analysis was used to analyze the correlations among mitochondrial functions, potential pathogenic factors, and immune cell infiltration status.

### Hub gene selection

Protein–protein interaction (PPI) analysis was conducted to select 20 genes from which to construct a diagnostic model. The STRING database and Cytoscape were used to evaluate the correlations among the Mito-DEGs. Genes with a degree ≥ 20 in the PPI network as visualized by Cytoscape were considered hub genes. Least absolute shrinkage and selection operator (LASSO) regression was used to select 7 hub genes for further single-cell RNA (scRNA) analysis.

### Construction and validation of the diagnostic model

A support vector machine (SVM) and datasets from GPL13158 and GPL570 (337 patients with UC and 97 HCs) were used to construct the model to distinguish UCs. This model was validated using 4 independent external validation datasets GSE 59071 (97 patients with UC and 11 HCs), GSE 97012 (7 patients with UC and 27 HCs), GSE 53306 (16 patients with UC and 12 HCs), and GSE 48958 (8 patients with UC and 7 HCs). The area under the receiver operating characteristic (AUROC) curve was used to assess the discrimination. A total of 1000 bootstrap resamplings were used to reduce the overfitting bias. The calibration curve and mean absolute error (MAE) were used to assess the calibration results. Decision curve analysis was used to assess clinical benefit. A clinical impact curve (CIC) was used to assess clinical impact.

### ScRNA sequencing data analysis

The GSE 214695 dataset (6 patients with UC and 6 HCs) was obtained from the GEO database. The raw data samples were screened according to the following conditions: (1) amount of RNA in a single cell ≥ 100 and ≤ 15000; and ([2) gene expression of mitochondria and ribosomes in the cells < 50%. The raw data were quality controlled and processed via the “Seurat” R package, and the batch effect was removed by the “harmony” R package. A total of 32110 cells were included for further analysis.

The “SingleR” and “clustree” R packages were used for automated data annotation and single-cell clustering. Uniform manifold approximation and projection (UMAP) [15] and t-distributed stochastic neighbor embedding (t-SNE) [16] were used for nonlinear dimensionality reduction visualization. The “CellChat” R package was used to analyze cell‒cell communication. The communication network of cell interactions was based on the communication probability. The “irGSEA”, “GSVA” and “GSEABase” R packages were used to determine the relationships between different cell types and signaling pathways.

### Tissue collection and preprocessing

Newly diagnosed and untreated UC patients were enrolled in the study. Between May 2023 and May 2024, UC colon tissues (n = 3, sampled on 02/08/2023, 05/10/2024, and 19/10/2023) and healthy colon tissues (n = 3, sampled on 05/06/2023, 12/06/2023, and 11/07/2023) were obtained from biopsies via colonoscopies (H290, Olympus Medical Systems, Tokyo, Japan) administered by the Second Affiliated Hospital of Harbin Medical University. The colon tissues were stored in 4% paraformaldehyde solution, dehydrated, embedded in paraffin, and sliced into 5 µm thick sections.

### Hematoxylin-eosin (H&E) staining

The tissue sections were heated, and deparaffinized, followed by H&E staining to assess inflammation in the colon tissue. The sections were observed and imaged using a BX51 microscope (Olympus, Tokyo, Japan).

### Immunohistochemistry (IHC) analysis

The tissue sections were boiled in ethylenediaminetetraacetic acid for antigen retrieval and endogenous peroxidase activity was suppressed by 3% hydrogen peroxide. The sections were incubated with anti-ACADM (Cat No: 67742–1-Ig, Proteintech, 1:150), and anti-ACADSB (Cat No: 13122–1-AP; Proteintech, 1:150) antibodies overnight at 4°C. On the following day, the sections were incubated at 37°C for 60 min with the corresponding secondary antibody, counterstained with hematoxylin, dehydrated, and fixed. The sections were observed and imaged using a BX51 microscope (Olympus, Tokyo, Japan), and the results were semiquantitatively analyzed with ImageJ (National Institutes of Health, Bethesda, USA).

### Immunofluorescence (IF) analysis

The tissue sections were blocked in 5% normal goat serum (abs933, Absin, Shanghai, China) for 60 min and incubated with anti-ACADM (Cat No: 67742–1-Ig, Proteintech, 1:150), and anti-ACADSB (Cat No: 13122–1-AP; Proteintech, 1:150) antibodies overnight. The sections were incubated at 37°C for 60 min with the corresponding secondary antibodies and stained with DAPI before observation via a fluorescence microscope (Nikon, Japan), and the results were semiquantitatively analyzed with ImageJ (National Institutes of Health, Bethesda, USA).

### Western blot (WB) analysis

The tissue sections were homogenized in lysis buffer containing protease inhibitors. The supernatants were collected after the lysates were centrifuged at 12,830 × g for 20 min at 4°C. A BCA protein concentration determination kit (Beyotime, P0012S) was used to assess protein concentrations. Processed protein samples were electrophoresed on SDS–PAGE gels and transferred onto PVDF membranes (Millipore, USA). The membranes were subsequently incubated at 4°C with anti-ACADM (Cat No: 67742–1-Ig, Proteintech, 1:1500) and anti-ACADSB (Cat No: 13122–1-AP; Proteintech, 1:1500) antibodies overnight after being blocked in 5% skim milk. The membranes were incubated at room temperature for 60 min with the corresponding secondary antibodies, and then visualized with an enhanced chemiluminescence solution. The results were semiquantitatively analyzed with ImageJ (National Institutes of Health, Bethesda, USA).

### Statistical analyses

Continuous variables are reported as medians with interquartile ranges. Categorical variables are reported as numbers and percentages. All the data were analyzed via R version 4.1.2 (R Foundation for Statistical Computing, Vienna, Austria), SPSS Modeler 18.0 software (SPSS Inc., Chicago, Illinois, USA) and the Statistical Package for Social Sciences 26.0 (SPSS, Inc. Chicago, Illinois, USA). A *P* value < 0.05 was considered to indicate statistical significance.

### Ethics statement

All the participants provided written informed consent for participation. No minors were recruited for the study. The study protocol was reviewed and approved by the Ethics Committee of the Second Affiliated Hospital of Harbin Medical University (ethics review batch number: KY2023−052).

## Results

### Mitochondrial factors and pathogenic pathways were enriched in the DEGs identified between patients with UC and HCs

**Fig 2:**
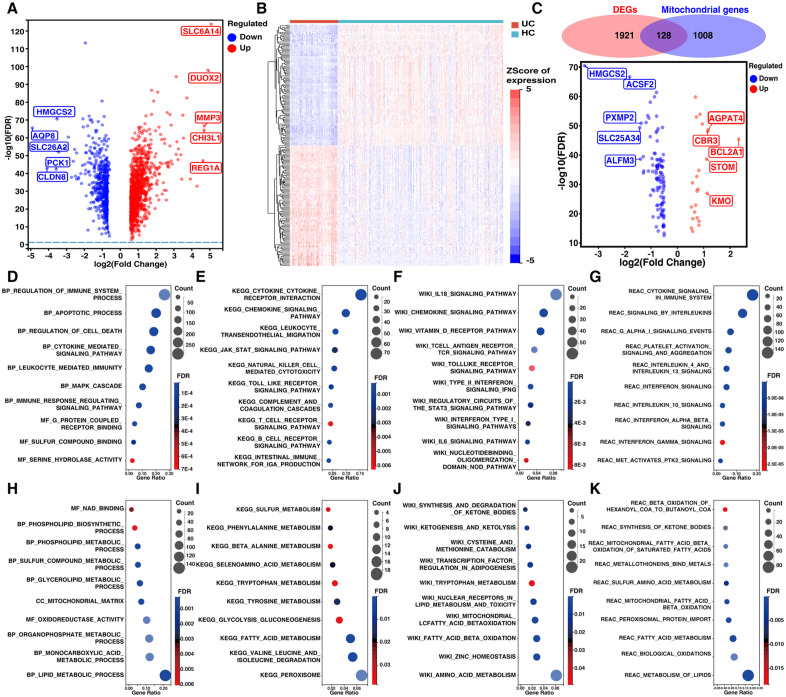
Mitochondrial alterations and pathogenic pathways identified among patients with UC and HCs. (A) Volcano plot showing 2049 DEGs between patients with UC and HCs. (B) Heatmap plot showing the top 100 upregulated and downregulated DEGs between patients with UC and HCs. (C) The Venn diagram and volcano plot of the 128 Mito-DEGs between patients with UC and HCs. (D–G) The upregulated Mito-DEGs among UCs and HCs were enriched in immune-related processes and pathogenic signaling pathways. (H–K) The downregulated Mito-DEGs among patients with UC and HCs were enriched in mitochondria-related structural and metabolic processes. (D, H) Enrichment results based on GO database; (E, I) Enrichment results based on KEGG database; (F, J) Enrichment results based on Wikipathway database; (G, K) Enrichment results based on REAC database.

A total of 2049 DEGs were identified between the UC patients and HCs (Fig 2A–B, S2 Table in S1 File), among with 128 Mito-DEGs were identified including 105 downregulated Mito-DEGs and 25 upregulated Mito-DEGs (Fig 2C, S3 Table, and S1 Fig in S1 File). Then, 2049 DEGs were used for enrichment analysis with the GO, KEGG, Wikipathway, and REAC databases. The enrichment analysis revealed that pathogenic signaling pathways and factors, including cytokines, chemokines, toll-like receptors, interferons, interleukins, and vitamin D and antigen receptors, were enriched among the 1238 upregulated DEGs (Fig 2D–G). Additionally, alterations in the structure and metabolic functions of mitochondria, including mitochondrial matrix, oxidation‒reduction, and the biosynthesis and metabolism of lipids, amino acids, carbohydrates, sulfur, and ketone bodies, were enriched in the 811 downregulated DEGs (Fig 2H–K).

### Development and validation of a diagnostic model for identifying UC using mitochondria-related genes

**Fig 3:**
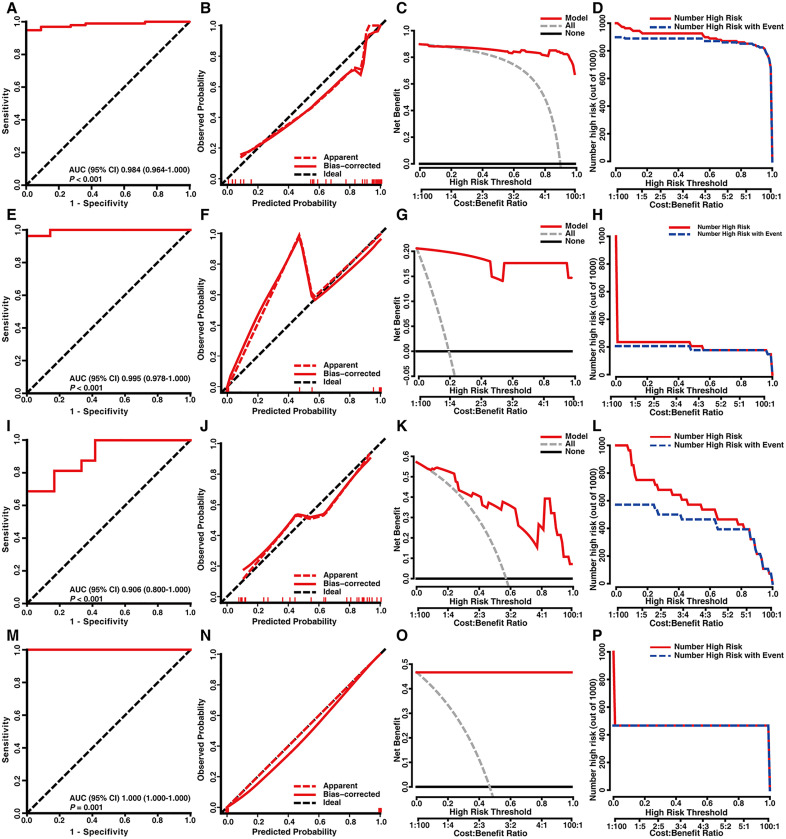
Mitochondria-related gene diagnostic model for distinguishing patients with UC from HCs in 4 external validation datasets. Model evaluation for 4 external validation sets: (A–D) GSE 59071; (E–H) GSE 97012; (I–L) GSE 53306; and (M–P) GSE 48958. The panels show: (A, E, I, M) ROC curves, (B, F, J, N) calibration curves, (C, G, K, O) decision curves, and (D, H, L, P) clinical impact curves.

20 Mito-DEGs whose degree was equal to or greater than 20 in the PPI network were selected (S2 Fig in S1 File), and SVM was used to construct the diagnostic model to distinguish patients with UC from HCs. This model achieved an AUROC of 0.981 (S3 FigA in S1 File) and an MAE of 0.025 (S3 FigB in S1 File). When an optimal cutoff value of 0.67 was applied, the model had a standard net benefit of 0.904 and a true positive rate of 0.964 S3 FigC–D, S4 Table in S1 File). The model was subsequently verified using 4 external datasets, which contained data from 128 patients with UC and 57 HCs. The AUROCs of the 4 validation sets were greater than 0.9. The model had a satisfactory discriminative capacity and demonstrated suitable calibration performance, clinical benefit and clinical impact (Fig 3, S4 Table in S1 File).

### Characterization of alterations in mitochondrial structure and function and pathogenic pathways between UC patient groups with high and low mitochondrial gene expression

**Fig 4:**
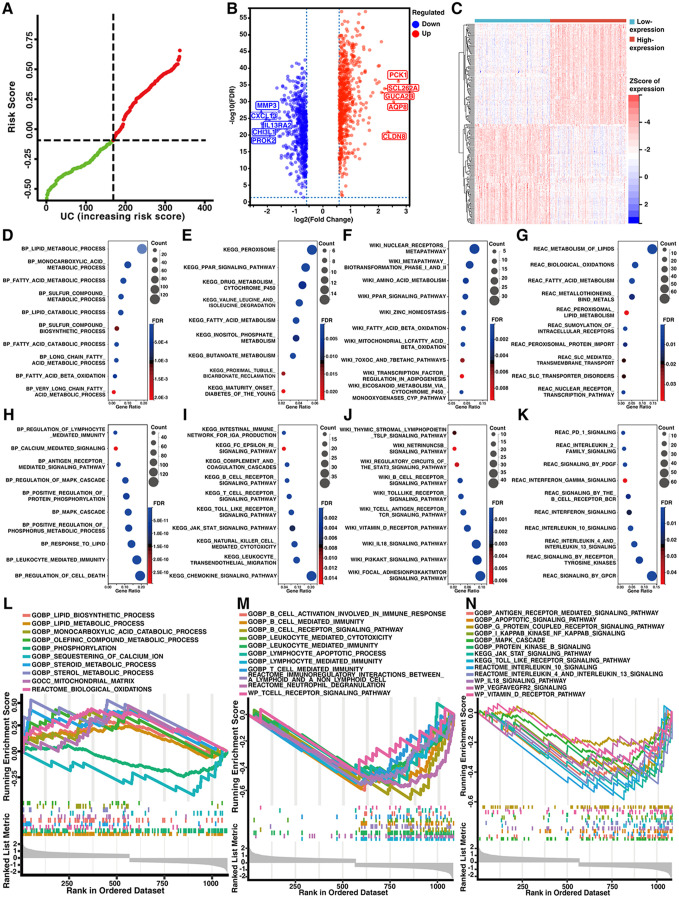
Mitochondrial dysfunction and the activation of pathogenic signaling pathways among UC patient groups with high and low mitochondrial gene expression. (A) GSVA risk scores based on mitochondrial gene expression in UC patients. (B) Volcano plot showing 1137 DEGs among UC patient groups with high and low mitochondrial gene expression. (C) Heatmap plot showed the top 100 upregulated and downregulated DEGs among UC patient groups with high and low mitochondrial gene expression. (D–G) The upregulated DEGs were enriched in metabolic processes and protective signaling pathways among the UC patient groups with high and low mitochondrial gene expression. (H–K) The downregulated DEGs were enriched in immune dysregulation and pathogenic signaling pathways among the UC patient groups with high and low mitochondrial gene expression. (D, H) Enrichment results of the GO database; (E, I) Enrichment results of the KEGG database; (F, J) Enrichment results of the Wikipathway database; (G, K) Enrichment results of the REAC database. GSEA of the (L) structure and function of mitochondria, and mitochondria-related metabolism, (M) immune response, and (N) pathogenic signaling pathways.

A total of 1136 mitochondria-related genes were used to calculate risk scores in UCs. According to the median, UC patients were divided into a high mitochondrial gene expression group (red, n = 168) and a low mitochondrial gene expression group (green, n = 169) (Fig 4A). A total of 1137 DEGs were identified, including 597 upregulated DEGs and 540 downregulated DEGs among UC patient groups with high and low mitochondrial gene expression (Fig 4B–C, S5 Table in S1 File). Critically, mitochondrial metabolic dysregulation was discovered as a core feature on the basis of enrichment analyses. The downregulated DEGs were enriched in genes related to mitochondrial energy metabolism, such as the TCA cycle [REACTOME_THE_CITRIC_ACID_TCA_CYCLE_AND_RESPIRATORY_ELECTRON_TRANSPORT; normalized enrichment score (NES) = 2.27, FDR = 7.42 × 10 ⁻ ^9^], the respiratory electron transport chain (GOBP_RESPIRATORY_ELECTRON_TRANSPORT_CHAIN; NES = 2.01, FDR = 2.71 × 10 ⁻ ⁶), and the ATP synthesis (GOBP_ATP_SYNTHESIS_COUPLED_ELECTRON_TRANSPORT; NES = 2.04, FDR = 4.67 × 10^−6^) phases of metabolism (S6 Table in S1 File). The upregulated DEGs were enriched in genes related to fatty acid oxidation (REACTOME_MITOCHONDRIAL_FATTY_ACID_BETA_OXIDATION; NES = 1.98, FDR = 1.26 × 10^−3^) (S6 Table in S1 File). Additionally, the upregulated DEGs involved metabolic processes and protective signaling pathways, including peroxisome proliferator-activated receptor (PPAR) and nuclear receptor signaling pathways (Fig 4D–G), whereas the downregulated DEGs involved immune dysregulation including immune system processes and complement cascades, and various pathogenic signaling pathways (Fig 4H–K). Moreover, GSEA revealed that genes related to mitochondrial structure and function (Fig 4L, S6 Table in S1 File), mitochondria-related metabolism (Fig 4L, S7 Table in S1 File), immune disorders caused by various immune cells (Fig 4M, S8 Table in S1 File) and several pathogenic signaling pathways (Fig 4N, S9 Table in S1 File) were enriched among the DEGs.

### Correlations between structural and functional factors of mitochondria and pathogenic factors

**Fig 5:**
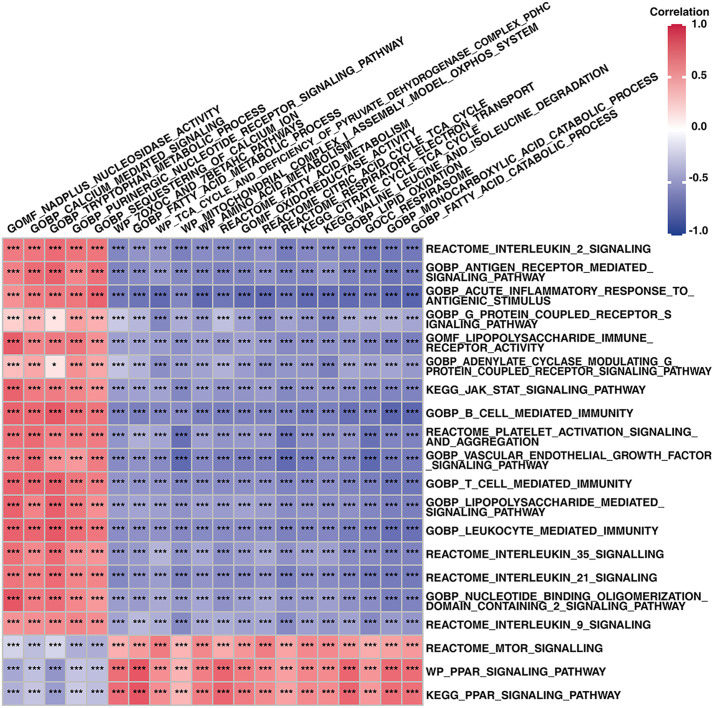
Correlations between mitochondrial dysfunction, immune activation and pathogenic pathways. *P < 0.05, **P < 0.01, ***P < 0.001.

We detected significant differences in the structural and functional factors of mitochondria and pathogenic factors between UC patients with high and low mitochondrial gene expression via GSVA scores (*P* < 0.05). We subsequently analyzed the correlations between mitochondrial gene expression and the structural and functional factors of mitochondria (S10 Table in S1 File) and between mitochondrial gene expression and pathogenic factors (S11 Table in S1 File). The correlations between the structural and functional factors of mitochondria and the pathogenic factors are shown in Fig 5.

### Immune cell infiltration analysis of UC patient groups with high and low mitochondrial gene expression

**Fig 6:**
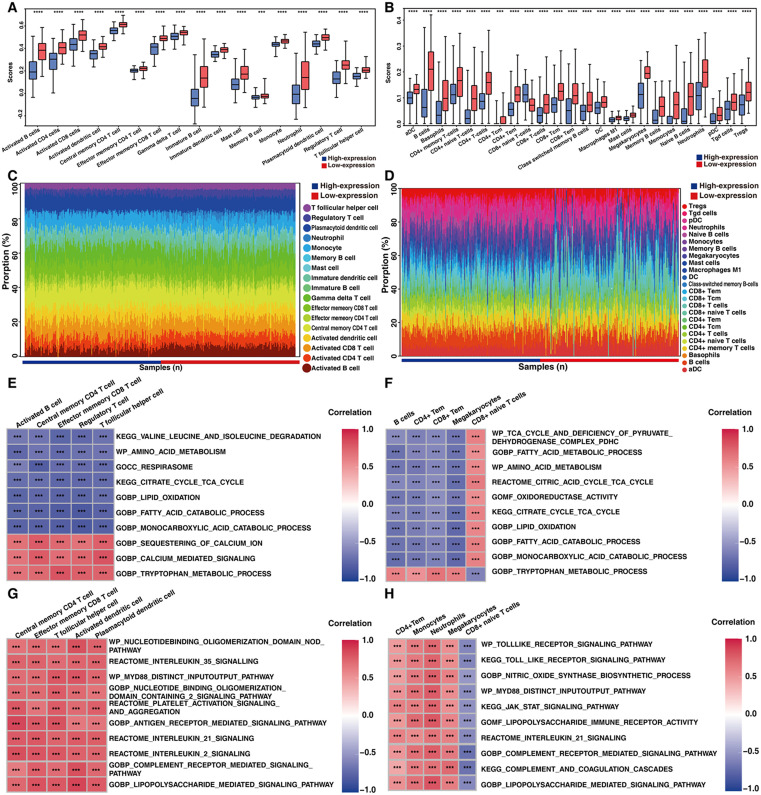
Immune microenvironment in UC patient groups with high and low mitochondrial gene expression. (A–B) Increased immune infiltration in UC patients with low mitochondrial gene expression. (C–D) Immune cell composition in UC patients with low mitochondrial gene expression. (E–F) Correlations among mitochondrial structure and mitochondria related functions and immune cells. (G–H) Correlations among UC related pathways and immune cells. A, C, E, and G were generated by ssGSEA. B, D, F and H were generated by xCell. *P < 0.05, **P < 0.01, ***P < 0.001.

The ssGSEA and xCell methods revealed significantly greater proportions of multiple immune cell types in UC patients with low mitochondrial gene expression than in those with high mitochondrial gene expression (Fig 6A–D, S12 Table, and S13 Table in S1 File). Notably, specific adaptive immune cell subsets, including B cells, CD4^+^ cells and CD8^+^ cells, were consistently among the most prominently enriched cell types identified by both analytical methods (Fig 6E–H). Correlation analysis revealed that increased immune infiltration in the low mitochondrial expression group was strongly associated with the respirasome, mitochondrial functions, and oxidoreductase activity within these immune cell clusters (Fig 6E–F). Furthermore, the infiltration levels of these immune cells, including CD4^+^ effector memory T cells (Tems), CD8^+^ Tems, monocytes, neutrophils, megakaryocytes, and activated and plasmacytoid dendritic cells, were significantly positively correlated with the activity of various signaling pathways (Fig 6G–H). An exception was CD8^+^ naïve T-cell infiltration, which was identified by xCell analysis and was negatively correlated with similar signaling pathways (Fig 6F, 6H).

### ScRNA analysis of the hub genes in UC patients and HCs

**Fig 7:**
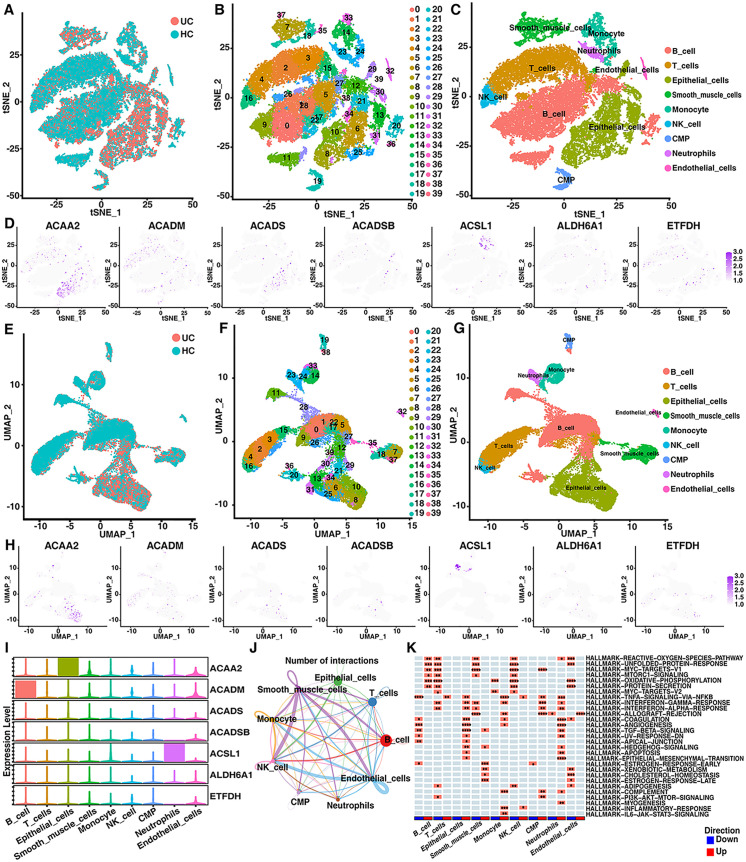
Single-cell transcriptomics of colon tissues from patients with UC and HCs. (A–D) T-SNE plots and (E–H) UMAP plots: (A, E) sample distribution, (B, F) cell clustering, (C, G) cell subpopulation, (D, H) hub gene localization. (I) Expression levels of the hub genes in the cell subpopulations. (J) Altered cell‒cell communication networks. (K) Pathway dysregulation in cell subpopulations. *P < 0.05, **P < 0.01, ***P < 0.001, ****P < 0.001.

A total of 12 scRNA samples from 6 UC patients and 6 HCs were analyzed (S14 Table in S1 File). t-SNE (Fig 7A) and UMAP (Fig 7E) analyses were used to visualize the scRNA data. t-SNE (Fig 7B) clustering and UMAP (Fig 7F) clustering revealed 32110 cells, which were clustered into 40 subgroups. LASSO regression was used to select the hub genes for diagnostic model construction, and 20 hub genes, including ACAA2, ACADM, ACADS, ACADSB, ACSL1, ALDH6A1 and ETFDH, were selected (S4 Fig in S1 File). Forty cell clusters were grouped using marker genes to determine cell distributions (Fig 7C,Fig 7G, and S5 Fig in S1 File). Information on the cell subpopulations is provided in S15 Table in S1 File. We also generated a feature plot (Fig 7D and 7H) and violin plot (Fig 7I) to visualize the gene expression of the selected 7 genes in all of the cell subpopulations. A cell‒cell communication network was constructed to calculate the communication probability, and the results revealed that immune cells interacted closely with intestinal tissue cells, including epithelial cells, endothelial cells and smooth muscle cells (Fig 7J). The regulation of different cell types by the “hallmark” signaling pathway is shown in Fig 7K, demonstrating that significant changes in this pathway affect both immune cells and intestinal tissue cells. A hallmark gene set was used to perform enrichment analysis on each type of immune cell separately. Pathways related to mitochondrial function, including the reactive oxygen species pathway, and the oxidative phosphorylation pathways, were enriched. Additionally, multiple UC disease signaling pathways including the MTORC1, NFκB, and TNFα signaling pathways, and several metabolic pathways such as cholesterol homeostasis, adipogenesis and xenobiotic metabolism are involved. These results reveal that immune cells may play crucial roles in linking colonic metabolism, mitochondrial function, and UC disease signaling pathways. Furthermore, we analyzed the composition ratios of different cell types in patients with UC and HCs (Fig S5 in S1 File). The results indicate that the proportions of various immune cells are elevated in patients with UC, which is consistent with the bulk-RNA immune infiltration results.

### Experimental validation of ACADM and ACADSB in patients with UC and HCs

**Fig 8:**
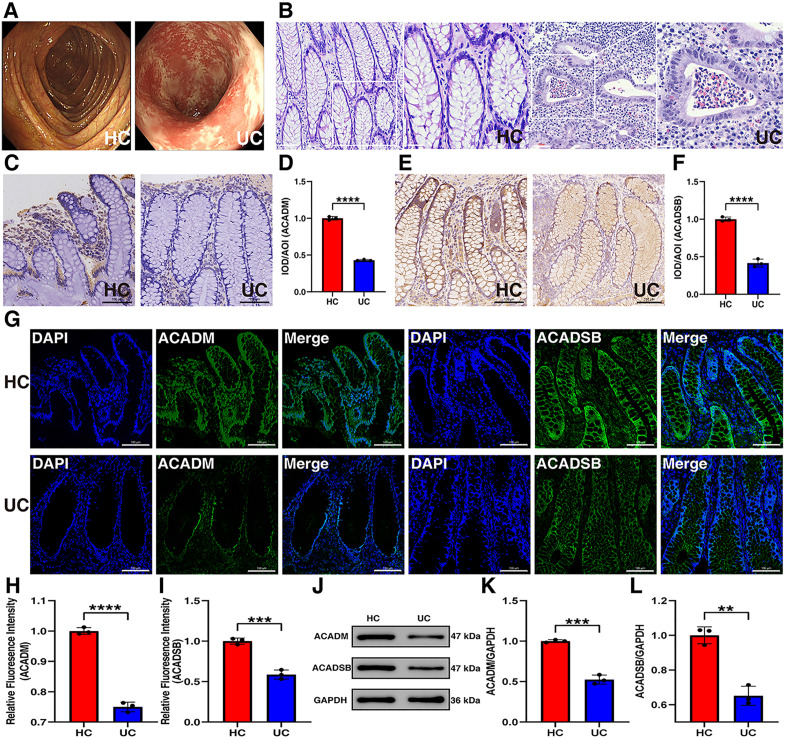
Experimental validation revealed that ACADSB and ACADM decreased in patients with UC. (A) Colonoscopy images. (B) H&E staining (scale bars = 60 μm). (C) Representative IHC image of ACADM in patients with UC and HCs (scale bars = 100 μm). (D) IHC shows reduced ACADM expression (1.000 ± 0.014 vs. 0.430 ± 0.005, *P* < 0.0001) with quantification in patients with UC patients. (E) Representative IHC image of ACADSB in patients with UC and HCs (scale bars = 100 μm). (F) IHC showing reduced ACADM expression (1.000 ± 0.016 vs. 0.416 ± 0.030, *P* < 0.0001) with quantification in UC patients. (G) Representative IF images of ACADM and ACADSB in patients with UC and HCs (scale bars = 100 μm). (H) IF shows reduced ACADM expression (1.000 ± 0.006 vs. 0.750 ± 0.009, *P* < 0.0001) with quantification in UC patients. (I) IF showing reduced ACADM expression (1.000 ± 0.022 vs. 0.587 ± 0.033, *P* < 0.001) with quantification in UC patients. (J) Representative WB images of ACADM and ACADSB in patients with UCs and HCs. (K) WB images showing reduced ACADM expression (1.000 ± 0.010 vs. 0.525 ± 0.032, *P* < 0.001) with quantification in UC patients. (L) WB images showing reduced ACADSB expression (1.000 ± 0.028 vs. 0.651 ± 0.032, *P* < 0.01) with quantification in UC patients. Data are presented as the mean ± SD; n = 3. Unpaired Student’s t-test was used for statistical analysis. **P < 0.01, ***P < 0.001, ****P < 0.0001.

Colonoscopy revealed ulcerations and the disappearance of the vascular pattern in the colon tissues of UC patients compared with those of HCs (Fig 8A), and the absence of crypt glands and inflammatory cell infiltration were observed in the colon tissue of UC patients (Fig 8B). Immunohistochemical (Fig 8C–F) and IF (Fig 8G–I) analyses revealed lower expression of ACADM and ACADSB in the colon tissues of UC patients compared with those of HCs. WB analysis revealed the same results (Fig 8J–L).

## Discussion

Our study aimed to identify mitochondrial-related genes related to immune cell infiltration that have diagnostic value for UC. Therefore, we first constructed and validated a UC diagnostic model based on mitochondria-related genes. A negative correlation was observed between mitochondria-related gene expression and both UC-associated pathogenic signaling pathways and immune cell infiltration. Our results indicated that mitochondria may be key protective organelles linking gut immune disorders, intestinal metabolic reprogramming, and activated signaling pathways within the mitochondria. Moreover, we identified hub genes and experimentally validated that compared with HCs, the expression of ACADM and ACADSB was decreased in the colon tissues of UC patients.

Mitochondrial metabolism and bioenergetics play important roles in maintaining intestinal mucosal barrier defense [17]. Mitochondrial damage leads to a decrease in ATP production, which is considered a main cause of intestinal dysfunction [2]. Mitochondria supply energy to cells by oxidizing carbohydrates, lipids and amino acids [18]. A recent study revealed that dysfunction of enterocyte mitochondria caused metabolic dysfunction and reduced ATP synthesis [19]. Another recent study revealed that an increase in short-chain fatty acids inhibited epithelial apoptosis via the Fas/Fasl pathway and suppressed colonic inflammation via toll-like receptor 4/NF-κB [20]. Mitochondrial metabolism generates various signaling molecules to alter the function of various immune cells, in which fatty acid oxidation (FAO) may be crucial [21]. In this study, enrichment analysis revealed that pathogenic signaling pathways were enriched in the upregulated DEGs and that structural factors and metabolic pathways were enriched in the downregulated DEGs between the patients with UC and HCs. More importantly, a diagnostic model was constructed using the identified Mito-DEGs, and its effectiveness in UC diagnosis was confirmed, which suggests that mitochondria play a vital role in UC pathogenesis. In addition, our enrichment analyses revealed that metabolic pathways, specifically the tricarboxylic acid cycle (TCA), lipid oxidation, fatty acid metabolism and amino acid metabolism were enriched among the upregulated DEGs and that ATP synthesis was enriched among the downregulated DEGs between UC patients with high and low mitochondrial gene expression levels. We propose that metabolic reprogramming may occur in the TCA cycle and FAO, leading to a reduction in ATP, increased reactive oxygen species (ROS), and subsequent energy deprivation in the epithelial cells of the colon.

**Fig 9:**
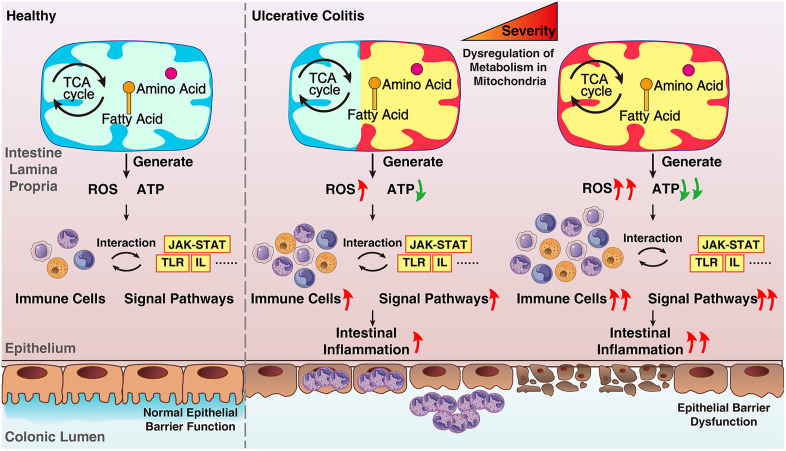
Proposed mechanism: Mitochondrial dysfunction drives UC pathogenesis.

One previous study revealed that perturbation of mitochondrial homeostasis caused by lipid and carbohydrate metabolic dysfunction regulated the innate immune response and aggravated inflammation in the intestinal epithelium in UC patients [22]. Moreover, ROS are considered vital mediators of mitochondrial signaling [18] and important components of the immune system [23]. The balance of mitochondrial ROS is essential for maintaining the integrity of the intestinal epithelium [24]. Damage to the electron transport chain and oxidative phosphorylation processes cause excessive ROS production, which exacerbates UC pathology [18,24]. Similarly, we found that the electron transport chain was enriched in the upregulated DEGs among UC patients with high mitochondrial gene expression. The immune response is considered a crucial player in the pathogenesis of UC [3]; however, its influence on mitochondria has not yet been revealed. Our study revealed that patients with UC with colonic mitochondrial dysfunction, which is characterized by low mitochondrial gene expression, a pathogenic cascade is triggered: altered mitochondrial metabolism elevates ROS levels and reduces ATP production, which synergistically promotes immune cell infiltration and activates disease-related signaling pathways. This crosstalk between dysregulated mitochondrial function in immune cells and colon epithelial and endothelial cells ultimately drives intestinal inflammation (Fig 9).

To verify the diagnostic value of the Mito-DEGs in UC, diagnostic models were constructed on the basis of 20 mitochondria-related genes using three popular methods (S3 Fig in S1 File). The SVM achieved an AUROC (95% CI) of 0.981 (0.970–0.992) (S3 FigA in S1 File), the decision tree model achieved an AUROC (95% CI) of 0.981 (0.969–0.994) (S3 FigE in S1 File), and the logistic regression model achieved an AUROC (95% CI) of 0.935 (0.910–0.960) (S3 FigI in S1 File). We also evaluated calibration (S3 FigB, F, J in S1 File), clinical benefit (S3 FigC, G, K in S1 File) and clinical impact (S3 FigD, H, L in S1 File) of these models. The AUROCs of all 3 methods were all greater than 0.900, which indicated that mitochondria-related genes were strongly different between patients with UC and HCs. SVMs, are widely used as clinical prediction models in binary classification [25] and are used to search for separation hyperplanes that maximize intervals in the feature space. In addition, SVMs have several advantages, such as great generalizability in the analysis of small sample sets [26]. We used 4 external validation datasets, which included data from 128 patients with UC and 57 HCs. The AUROCs of the SVM model were all greater than 0.900 with these datasets, demonstrating the effectiveness of the SVM model distinguishing patients with UC from HCs (Fig 3 and S4 Table in S1 File).

Our integrated analysis of bulk-seq and scRNA-seq data elucidates the relationship among mitochondrial alteration in each cell type and colon tissue. While bulk RNA-seq effectively identifies disease-associated gene signatures, it conflates changes in gene expression with changes in cellular abundance. ScRNA-seq data from matched tissues can help mitigate these limitations and validate the major cellular sources of the bulk DEGs. This cell-type-specific expression pattern indicates that the differential expression observed in bulk sequencing arises from a combination of true transcriptional changes within specific cell types and alterations in immune infiltration and potential loss of epithelial integrity. Our scRNA-seq analysis effectively distinguished these two factors, confirming that the key mitochondrial alterations in UC are both a cause and a consequence of the disrupted cellular ecosystem in the colonic mucosa (S6 Fig in S1 File). Although computational deconvolution methods are powerful for inferring cellular proportions [27], their reliance on predefined gene signatures and inability to capture novel or disease-specific cell states are limitations.

FAO plays an important role in UC. A previous study reported that FAO is essential for M2 macrophage polarization and intestinal repair and remodeling [28]. Therefore, we chose ACADM and ACADSB as our genes of interest owing to their important role in the breakdown of fatty acids.

ACADM, an acyl-CoA dehydrogenase, catalyzes mitochondrial fatty acid beta-oxidation and is differentially expressed in hepatocellular carcinoma [29], cholangiocarcinoma [30], and coronary microvascular dysfunction [31]. In a recent study multiomics data were used to provied tier 1 evidence that lower of ACADM expression (OR 0.67, 95% CI 0.55–0.83) was associated with UC risk, which was similar to our study results [32]. ACADSB catalyzes the conversion of fatty acyl-CoA thioesters to trans-2-enoyl-CoA, whose expression is decreased in several tumors. Studies have demonstrated that the expression of ACADSB is negatively correlated with colorectal cancer stage [33]; ACADSB expression is also negatively correlated with stage and grade in clear cell renal cell carcinoma and is an independent factor of overall survival [34]. Liver cancer patients with low ACADSB expression have poor survival outcomes [35]. However, no studies have investigated the change in ACADSB expression in patients with UC. On the basis of the bioinformatics analysis, we perfomed IHC, IF and WB experiments to verify the decreased ACADSB expression in UC patients. ScRNA analysis revealed that ACADM was predominantly expressed in B cells, and that ACADSB was predominantly expressed in epithelial cells. ACADM and ACADSB are key genes in FAO, which supplies ATP for epithelial barrier homeostasis and is important for metabolic progression in UC. We propose that the decrease in ACADM and ACADSB expression levels weakens the energy supply and damages the mucosal integrity. However, whether ACADM and ACADSB influence mitochondrial function by affecting lipid metabolism and thereby participate in the pathogenesis of UC remains unclear. This issue will be the focus of our future research.

Although our study revealed novel insights, it still has several limitations. First, our data were obtained from the GEO database. Although we verified the expression of the hub genes ACADM and ACADSB in our clinical samples, our sample size was limited and more clinical information, such as severity, is needed. In addition, we need to verify the generalizability of our model and correlate hub mitochondria-related genes with clinical severity in future studies. Second, 20 hub genes were used to construct a diagnostic model for UC. We validated the discrimination, calibration, clinical benefit and clinical impact in the validation sets but did not verify all 20 genes experimentally. Third, the role of mitochondria in UC pathogenesis is complex, and we still need to explore the specific mechanism mediated by ACADM and ACADSB using rescue experiments in specific cells in future research. Fourth, we have not yet performed sufficient experiments, such as flow cytometry and immune phenotyping analysis, on the changes in the expression of each type of immune cell in UC.

## Conclusions

Our study revealed that mitochondria play important roles in UC pathogenesis by altering energy metabolism, promoting immune disorders and activating pathogenic signaling pathways. Our model, which is based on mitochondria-related genes showed excellent discrimination, calibration, clinical benefit and clinical impact in UC diagnosis. Moreover, ACADM and ACADSB expression was significantly downregulated in the colon tissues of UC patients, suggesting that these genes may be potential therapeutic targets for UC.

## Supporting information

S1 Data Raw Data. (ZIP)

S1 File **S1 Fig.** ROC curves and expression level of hub genes. (A) ROC curves of 9 hub genes distinguish UCs from HCs. Expression levels of (B) ACAA2, (C) ACADM, (D) ACADS, (E) ACADSB, (F) ACSL1, (G) ALDH6A1, and (H) ETFDH. ROC, receiver operating characteristic. **S2 Fig.** PPI network of Mito-DEGs. **S3 Fig.** The evaluation of diagnostic model based on mitochondria-related genes to distinguish ulcerative colitis from healthy controls in training set. (A-D) presents model evaluation based on SVM. (E-H) presents model evaluation for decision tree. (I-L) presents model evaluation for logistic regression. (A, E, I) ROC curves. (B, F, J) Calibration curves. Smoothed lines fit to the curve and vertical bar illustrates the distribution of predictions. (C, G, K) Decision curves. (D, H, L) Clinical impact curves. **S4 Fig.** LASSO regression to select hub genes. (A) Cross validation for tuning parameter selection. (B) LASSO coefficient profiles of 7 mitochondria-related genes. **S5 Fig.** Cell proportion of 9 type of cells in UC and HC samples. **S6 Fig.** Venn plots illustrating the overlap among mitochondria-related genes, bulk-RNA sequencing differentially expressed genes (bulk-DEGs), and cell type-specific DEGs across nine distinct cell populations. **S1 Table.** RNA sequencing data enrolled in study. GEO, Gene Expression Omnibus; HC, healthy control; UC, ulcerative colitis. **S2 Table.** DEGs were identified between UCs and HCs. **S3 Table.** The expression of Mito-DEGs in UCs and HCs. The hub genes are highlighted in red font. **S4 Table.** The assessment of diagnostic model in training and validation sets. **S5 Table.** DEGs were identified between high and low mitochondrial gene expression in UC. **S6 Table.** GSEA enrichment analysis in mitochondrial structure and function in high-expression mitochondria UC compared to low-expression mitochondria UC. **S7 Table.** GSEA enrichment analysis in mitochondrial related metabolism in high-expression mitochondria UC compared to low-expression mitochondria UC. **S8 Table.** GSEA enrichment analysis in immune response in high-expression mitochondria UC compared to low-expression mitochondria UC. **S9 Table.** GSEA enrichment analysis in signaling pathways in high-expression mitochondria UC compared to low-expression mitochondria UC. **S10 Table.** Correlation between mitochondria expression and the structure and function of mitochondria. **S11 Table.** Correlation between mitochondria expression and pathogenic factors. **S12 Table.** The expression of immune cells in high/low expression mitochondria UC patients based on ssGSEA. **S13 Table.** The expression of immune cells in high/low expression mitochondria UC patients based on xCell. **S14 Table.** The detail of samples for Sc-RNA seq analysis. **S15 Table.** Information of cell subpopulation.
(ZIP)

S1 Code Code. (ZIP)
