# Supplementary material for: Unveiling and verification of mitochondria-related genes as potential diagnostic biomarkers in ulcerative colitis based on bioinformatics analysis and experimental validation
Source: PLoS One. 2025 Nov 4;20(11):e0336224. doi: 10.1371/journal.pone.0336224 (PMC12585056; doi:10.1371/journal.pone.0336224)
Supplement: S1 File — S1 Fig. ROC curves and expression level of hub genes. (A) ROC curves of 9 hub genes distinguish UCs from HCs. Expression levels of (B) ACAA2, (C) ACADM, (D) ACADS, (E) ACADSB, (F) ACSL1, (G) ALDH6A1, and (H) ETFDH. ROC, receiver operating characteristic. S2 Fig. PPI network of Mito-DEGs. S3 Fig. The evaluation of diagnostic model based on mitochondria-related genes to distinguish ulcerative colitis from healthy controls in training set. (A-D) presents model evaluation based on SVM. (E-H) presents model evaluation for decision tree. (I-L) presents model evaluation for logistic regression. (A, E, I) ROC curves. (B, F, J) Calibration curves. Smoothed lines fit to the curve and vertical bar illustrates the distribution of predictions. (C, G, K) Decision curves. (D, H, L) Clinical impact curves. S4 Fig. LASSO regression to select hub genes. (A) Cross validation for tuning parameter selection. (B) LASSO coefficient profiles of 7 mitochondria-related genes. S5 Fig. Cell proportion of 9 type of cells in UC and HC samples. S6 Fig. Venn plots illustrating the overlap among mitochondria-related genes, bulk-RNA sequencing differentially expressed genes (bulk-DEGs), and cell type-specific DEGs across nine distinct cell populations. S1 Table. RNA sequencing data enrolled in study. GEO, Gene Expression Omnibus; HC, healthy control; UC, ulcerative colitis. S2 Table. DEGs were identified between UCs and HCs. S3 Table. The expression of Mito-DEGs in UCs and HCs. The hub genes are highlighted in red font. S4 Table. The assessment of diagnostic model in training and validation sets. S5 Table. DEGs were identified between high and low mitochondrial gene expression in UC. S6 Table. GSEA enrichment analysis in mitochondrial structure and function in high-expression mitochondria UC compared to low-expression mitochondria UC. S7 Table. GSEA enrichment analysis in mitochondrial related metabolism in high-expression mitochondria UC compared to low-expression mitochondria UC. S8 Table. GSEA en [file pone.0336224.s002.zip › Supporting Information/S Table/S1 Table.docx]

**S1 Table. RNA sequencing data enrolled in study.**

| GEO accession | Platform | Subgroup | Number of samples | |
| --- | --- | --- | --- | --- |
|  |  |  | UCs | HCs |
| GSE 92415 | GPL13158 | Training set | 162 | 21 |
| GSE 87473 | GPL13158 | Training set | 87 | 21 |
| GSE 47908 | GPL570 | Training set | 39 | 15 |
| GSE 38713 | GPL570 | Training set | 15 | 13 |
| GSE 36807 | GPL570 | Training set | 15 | 7 |
| GSE 22619 | GPL570 | Training set | 10 | 10 |
| GSE 13367 | GPL570 | Training set | 9 | 10 |
| GSE 59071 | GPL6244 | Validation set 1 | 97 | 11 |
| GSE 97012 | GPL13607 | Validation set 2 | 7 | 27 |
| GSE 63306 | GPL4091 | Validation set 3 | 16 | 12 |
| GSE 48958 | GPL6244 | Validation set 4 | 8 | 7 |

GEO, Gene Expression Omnibus; HC, healthy control; UC, ulcerative colitis.
